# Rust-accelerated powder X-ray diffraction simulation for high-throughput and machine-learning-driven materials science

**DOI:** 10.1107/S1600576726005273

**Published:** 2026-07-22

**Authors:** Miroslav Lebeda, Jan Drahokoupil, Petr Veřtát, Petr Vlčák

**Affiliations:** a Faculty of Mechanical Engineering Czech Technical University in Prague Technická 4 16607Prague 6 Czechia; b Faculty of Nuclear Sciences and Physical Engineering Czech Technical University in Prague Trojanova 339/13 12000Prague 2 Czechia; c Institute of Physics of the Czech Academy of Sciences Na Slovance 2 18200Prague 8 Czechia; Goa University, India

**Keywords:** powder X-ray diffraction, Rust, Python, *pymatgen* software, high-throughput materials characterization

## Abstract

High-throughput powder X-ray diffraction (XRD) simulations are a key prerequisite for generating large datasets used in the development of machine-learning models for XRD-based materials analysis. However, the widely used *pymatgen* powder XRD calculator, implemented entirely in Python, can be computationally inefficient for large-scale workloads, limiting throughput. We present *XRD-Rust*, a Rust-accelerated implementation of the *pymatgen* powder XRD calculator that maintains compatibility with the existing Python-based workflows. The method retains *pymatgen* for crystal structure handling, while reimplementing the computationally intensive parts of the XRD calculation in the Rust language, with optional further acceleration via single instruction, multiple data (SIMD) vectorization and multi-threaded execution across reflections using the *Rayon* library. Performance benchmarking on two large crystallographic datasets, the Materials Cloud Three-Dimensional Structure Database (MC3D, 33142 structures) and the Crystallography Open Database (COD, 515181 structures), demonstrates substantial speedups. For the MC3D, *XRD-Rust* achieves a median serial SIMD speedup of 10.8× [median absolute deviation (MAD) of 1.8×], increasing to 15.1× (MAD 3.8×) with eight-thread parallel execution, with a maximum runtime reduction from 40.5 s to 0.9 s. For the COD, the median serial SIMD acceleration reaches 10.7× (MAD 4.2×), while eight-thread parallel execution yields 19.5× (MAD 10.0×), with a maximum reduction from 1437 to 1 min. SIMD vectorization alone provides additional performance gains ranging from a few percent to several tens of percent, depending on the workload and execution mode. Overall, these benchmarks demonstrate that *XRD-Rust* significantly accelerates powder XRD simulations compared with the original *pymatgen* implementation, enabling efficient high-throughput dataset generation and improving performance in interactive diffraction analysis applications.

## Abstract 2

*XRD-Rust* is a Rust-accelerated reimplementation of the computational core of the *pymatgen* powder X-ray diffraction calculator that preserves full Python workflow compatibility while significantly improving performance for large-scale simulations. Benchmarks on large crystallographic databases show average speedups of 4–6× and peak gains of up to 719×, enabling efficient high-throughput dataset generation and faster interactive diffraction analysis.

## 1. Introduction

Powder X-ray diffraction (XRD) is one of the most widely used techniques for materials characterization. Computational simulation of powder XRD patterns from known crystal structures plays a central role in phase identification, structure validation and construction of reference databases. In recent years, simulated diffraction patterns have also become an essential component of data-driven materials science, particularly for training and validating machine-learning (ML) models for automated XRD analysis (Surdu & Győrgy, 2023 ▸).

A growing number of reports have demonstrated that ML models can perform XRD-related tasks such as crystal system classification (Salgado *et al.*, 2023 ▸; Lolla *et al.*, 2022 ▸; Li *et al.*, 2025 ▸), phase identification (Lee *et al.*, 2020 ▸; Szymanski *et al.*, 2021 ▸), qualitative phase analysis (Simonnet *et al.*, 2024 ▸) and ML-assisted Rietveld refinement (Guo *et al.*, 2024 ▸). These models typically rely on very large synthetic training datasets generated from experimentally known or theoretically predicted crystal structures, often comprising millions of diffraction patterns modified with experimental effects such as peak broadening, noise and preferred orientation. Similar computational demands arise in generative models for crystal structure prediction (Johansen *et al.*, 2025 ▸) and in active-learning experimental workflows (Szymanski *et al.*, 2023 ▸), where diffraction patterns must be calculated repeatedly during optimization. The crystal structures used to generate such datasets are commonly obtained from large crystallographic databases, for instance from the Materials Project (publicly available; Jain *et al.*, 2013 ▸), the Crystallography Open Database (COD, publicly available; Gražulis *et al.*, 2009 ▸), the Open Quantum Materials Database (publicly available; Saal *et al.*, 2013 ▸), the Materials Cloud Three-Dimensional Structure Database (MC3D, publicly available; Huber *et al.*, 2026 ▸) or the Inorganic Crystal Structure Database (licensed; Hellenbrandt, 2004 ▸).

The generation of such large datasets imposes demands on the efficiency of powder XRD simulation. Within widely employed Python-based workflows, an established tool is the XRD calculator implemented in the *pymatgen* library (MIT licence; Ong *et al.*, 2013 ▸). This implementation incorporates kinematic diffraction theory, atomic scattering factors, Lorentz polarization (Lp) corrections and optional thermal effects. Due to its availability in the *pymatgen* Python package, it can be easily employed in the workflow for high-throughput calculations of diffraction patterns. As a result, it has become one of the standards for Python-based crystallographic analysis. However, since *pymatgen*’s XRD calculator is implemented in pure Python, its performance can become a significant bottleneck in high-throughput and ML-oriented workflows, particularly for large unit cells, low-symmetry structures or the batch processing of a large number of structures.

Besides Python-based *pymatgen*, several established crystallographic software packages provide functionality for simulating powder XRD patterns. These include publicly available tools such as *Profex* (Doebelin & Kleeberg, 2015 ▸), *GSAS-II* (Toby & Von Dreele, 2013 ▸), *Maud* (Lutterotti, 2000 ▸) and *JANA2020* (Petříček *et al.*, 2023 ▸), as well as licensed commercial software including Rigaku *SmartLab Studio*, PANalytical *HighScore* (Degen *et al.*, 2014 ▸) and *TOPAS* (Coelho, 2018 ▸). Visualization-oriented tools such as the publicly available *VESTA* (Momma & Izumi, 2011 ▸) or the licensed software *Diamond* (Brandenburg, 2014 ▸) are also commonly employed. While these packages are widely used for diffraction analysis and simulation, they are primarily designed for interactive or file-based workflows and offer limited integration with Python-based data pipelines and ML frameworks.

The closest alternatives to *pymatgen*’s XRD calculator among Python-based implementations appear to be *Dans Diffraction* (https://github.com/DanPorter/Dans_Diffraction; Porter, 2020 ▸) and *Xrayutilities* (Kriegner *et al.*, 2013 ▸). *Dans Diffraction* seems more suitable for smaller calculations rather than large-scale workflows because it prints information to the console and is fully Python based like *pymatgen*. On the other hand, *Xrayutilities* is a Python package with several routines written in C, offering a significantly faster alternative to *pymatgen*. However, since *pymatgen* is currently one of the most widely employed packages in materials science, providing a faster alternative that is directly compatible with *pymatgen* notation is highly beneficial for already implemented workflows or for researchers already familiar with it.

As an example, in *XRDlicious* (https://xrdlicious.com; Lebeda *et al.*, 2025 ▸), our recently developed browser-based interactive application for powder diffraction calculation that employs *pymatgen*, a noticeable performance reduction is observed when processing for instance large organic crystal structures containing hundreds of atoms, particularly when diffraction patterns from multiple structures are uploaded simultaneously for comparison. In such cases, the time required to generate diffraction patterns can increase to tens of seconds or more, significantly impairing the user experience. This further confirms the need for more optimized implementations that combine the performance, usability and interoperability of Python-based workflows while notably reducing computation times.

To address these limitations, we present *XRD-Rust*, a Rust-accelerated implementation of the existing *pymatgen* powder XRD calculator. The approach does not introduce new diffraction algorithms but focuses on implementation-level performance optimization while maintaining full compatibility with existing *pymatgen*-based workflows. Rust is a compiled programming language designed for high-performance execution of computationally intensive tasks (Klabnik & Nichols, 2023 ▸; Mroczek *et al.*, 2025 ▸). Our approach retains *pymatgen*’s functionality for crystal structure handling, while accelerating the performance-critical components of the calculation using Rust. The performance of the Rust-accelerated implementation is benchmarked on two large crystal structure databases, MC3D and COD.

For the MC3D dataset, a serial Rust-accelerated calculator with single instruction, multiple data (SIMD) vectorization achieves a median speedup of 10.5× with a median absolute deviation (MAD) of 1.8×. The maximum calculation time reduction was from 40.5 to 0.9 s. For the COD dataset, the corresponding median speedup is 10.7× (MAD 4.2×) and the maximum time reduction is from 1437 to 1 min. Parallelization with the *Rayon* library on eight threads with SIMD further improves the median speedup to 14.6× (MAD 3.8×) for the MC3D and to 19.5× (MAD 10.0×) for the COD.

While *XRD-Rust* is not intended to replace established highly optimized crystallographic programs implemented in compiled languages such as C, C++ or Fortran, it significantly improves the computational efficiency of powder XRD simulations within the Python ecosystem, particularly in workflows based on *pymatgen*. By combining the flexibility of Python and its integration with ML frameworks with Rust-based acceleration, the proposed approach occupies a complementary position between ease of use and high performance. Ultimately, this enables scalable high-throughput simulations while preserving seamless compatibility with existing *pymatgen* workflows

## 2. Implementation details

Rather than completely rewriting *pymatgen*’s XRD calculator, we adopted a targeted optimization strategy that combines Python and Rust. The Python *pymatgen* layer is used for crystallographic operations with low computational cost, including parsing crystal structure data and applying space-group symmetry. The performance-critical numerical components were reimplemented in Rust. These include the generation of reciprocal-lattice points within the physically accessible sphere, the nested loops for structure-factor evaluation by summing atomic contributions for each reflection, pairwise operations for grouping symmetry-equivalent reflections, the application of geometric and physical correction factors (Lorentz polarization and Debye–Waller), and the merging of reflections within specified angular tolerances. Additionally, multi-threading across reflections is provided via the *Rayon* data-parallelism library (https://github.com/rayon-rs/rayon), with the number of threads configurable by the user. The structure-factor inner loop is further accelerated using SIMD vectorization via the *wide* package (https://github.com/Lokathor/wide).

The Rust-accelerated implementation does not apply any built-in peak filtering and instead returns the complete list of calculated reflections. Optional filtering can be performed subsequently as a post-processing step if desired. By contrast, the original *pymatgen* implementation automatically removes reflections with intensities below 0.1% of the maximum peak intensity. This behaviour is not exposed as a user-configurable parameter and can only be changed by modifying the *pymatgen* package source code. Because this filtering step is computationally inexpensive (tens of thousands of reflections typically requiring only a few milliseconds to determine), it does not affect performance comparisons between the two approaches.

**Figure 1:**
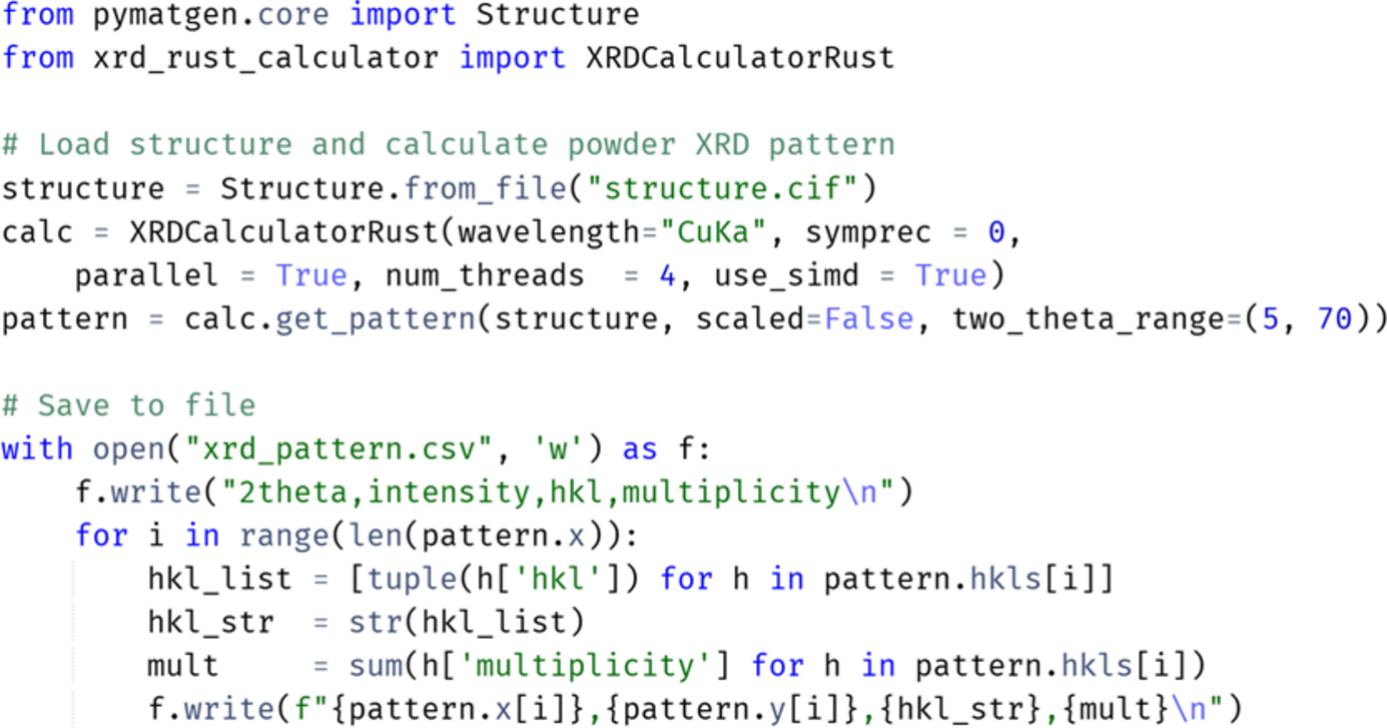
Minimal Python script demonstrating powder XRD pattern calculation using the Rust-accelerated calculator with four-thread parallelization via the *Rayon* library and SIMD vectorization.

The Rust-accelerated implementation interfaces with Python through *PyO3* (https://github.com/PyO3/pyo3), which provides bindings between Rust and Python. The Rust code is compiled into a platform-specific shared library using *Maturin* (https://github.com/PyO3/maturin), a build system designed for packaging Rust–Python extensions. The compiled libraries can be imported like standard Python modules, allowing Rust functions to be called directly from Python while *PyO3* automatically handles data conversion between the two languages. The interface accepts standard *pymatgen* structure objects and returns diffraction patterns in the same format as the original *pymatgen* implementation, requiring no major modifications to existing workflows. A minimal example script demonstrating powder XRD pattern calculation with the Rust-accelerated package is shown in Fig. 1 ▸.

## 3. Numerical validation

**Figure 2:**
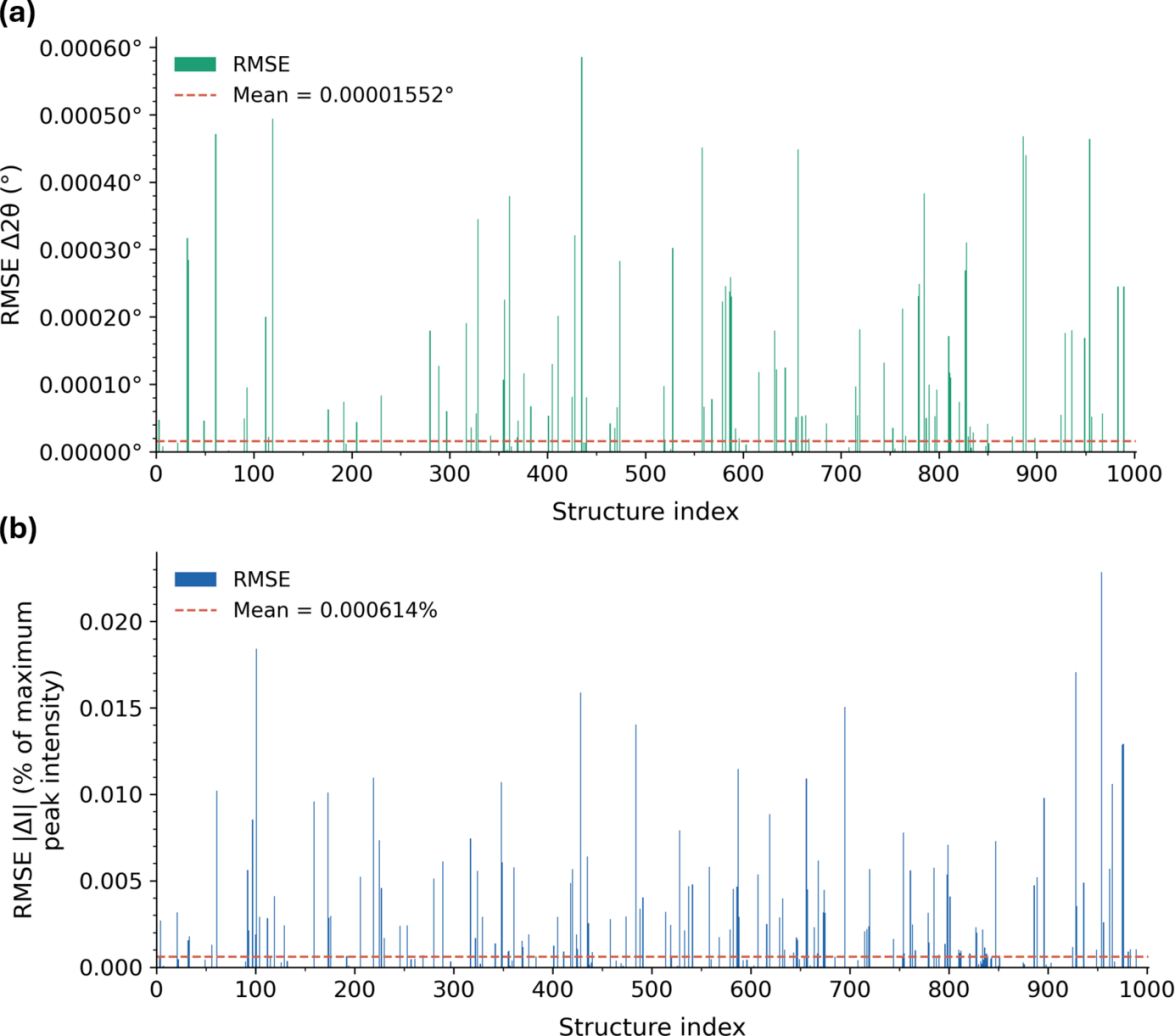
Numerical comparison of (*a*) peak positions and (*b*) intensities between *XRD-Rust* and the original *pymatgen* XRD calculator across 1000 COD structures, each having between 500 and 1500 atoms (total of 8638808 reflections, 2θ within 2–60°, Mo radiation).

To validate the numerical consistency of *XRD-Rust* with the original *pymatgen* implementation, we compared peak positions and intensities up to eight decimal places across the first 1000 sorted structures from the COD containing between 500 and 1500 atoms (Fig. 2 ▸). For each structure, we computed the root mean-square error (RMSE), followed by an overall RMSE across all structures, comprising a total of 8638808 reflections. The peak positions calculated by *XRD-Rust* show excellent agreement, with a mean RMSE of 0.00001552° (maximum RMSE of 0.00058562°), well below typical experi­mental uncertainty. The peak intensities, normalized to a 0–100 scale, exhibit a mean RMSE of 0.000614% relative to the maximum peak intensity. No reflection showed an intensity deviation exceeding 1% of the maximum intensity, and the worst-case RMSE across all structures remained as low as ∼0.023%. These results demonstrate that *XRD-Rust* reproduces*pymatgen* outputs with high numerical fidelity, with no physically meaningful deviations in peak positions or intensities.

## 4. Performance

The performance of the Rust-accelerated XRD calculator was benchmarked against the original *pymatgen* implementation using two crystal structure databases: MC3D (33142 structures as of 29/12/2025) and COD (521900 structures, revision 297631). Powder XRD patterns were calculated over a 2–60° 2θ range using Mo *K*α radiation. Four execution configurations of the Rust-accelerated implementation were evaluated: serial execution without SIMD (reflecting primarily the performance gain from transitioning from Python to Rust), serial execution with SIMD vectorization, eight-thread parallel execution using the *Rayon* library without SIMD and eight-thread parallel execution with SIMD.

### 4.1. Performance benchmarking on the MC3D

**Figure 3:**
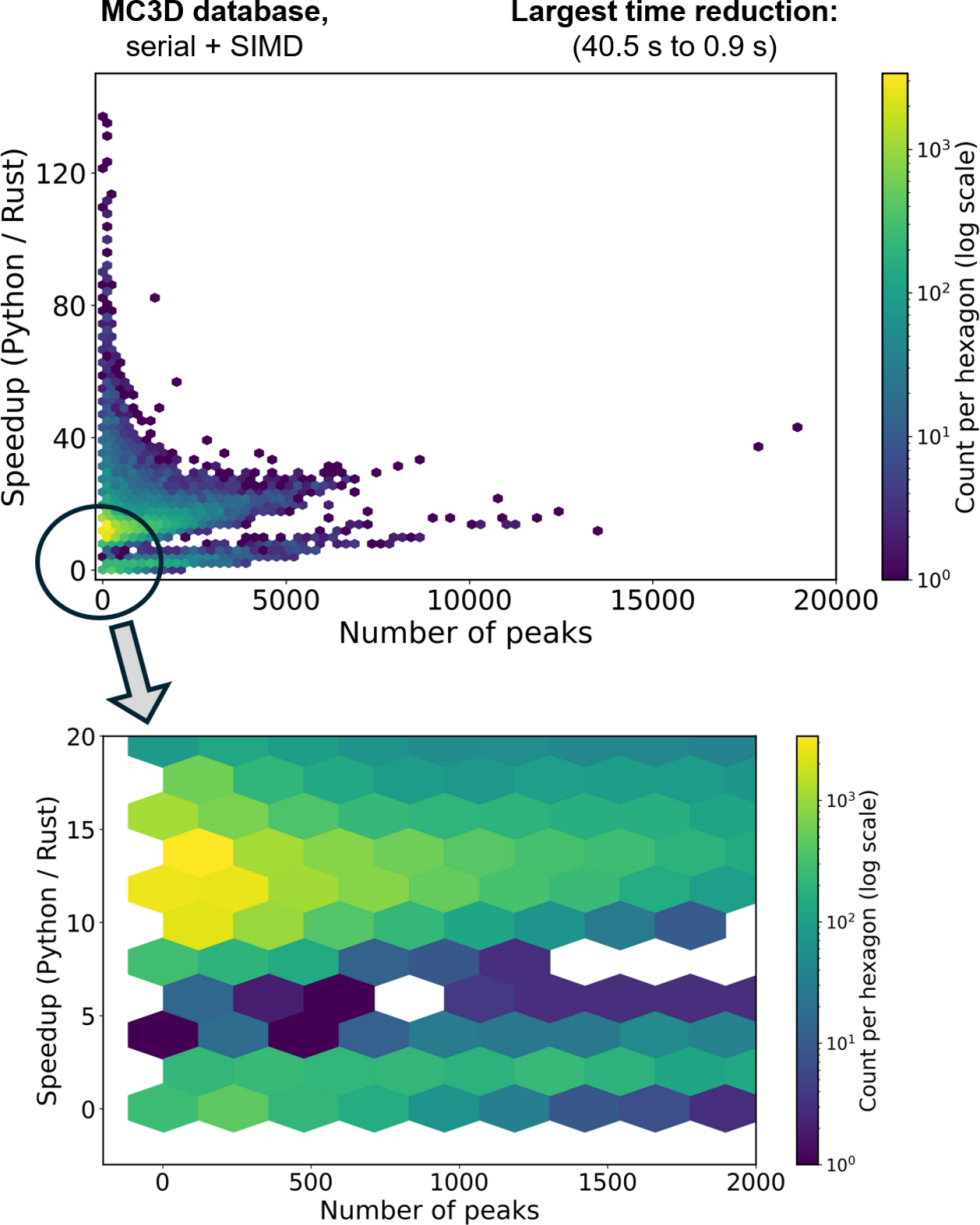
Performance comparison between the Rust-accelerated XRD calculator with serial execution and SIMD vectorization and the original *pymatgen* implementation. Benchmarks were performed on 33142 crystal structures from the MC3D, with powder XRD patterns computed in the 2–60° 2θ range using Mo radiation. The median speedup is 12.6× (MAD 2.0×).

For serial execution with SIMD vectorization, the Rust implementation achieved a median speedup of 12.6× (MAD 2.0×) relative to the original *pymatgen* implementation across the MC3D dataset (Fig. 3 ▸). In 1138 cases (∼3.4% of structures), performance was comparable with or slightly slower than *pymatgen*. These cases involved only a small number of reflections, with an average runtime of ∼0.14 s using the Rust-accelerated version versus ∼0.08 s for the original *pymatgen*, making the difference practically negligible. The largest runtime reduction was observed for structure mc3d-80944, for which a diffraction pattern containing 18948 reflections required 40.5 s with *pymatgen* but only 0.9 s with the Rust implementation.

**Figure 4:**
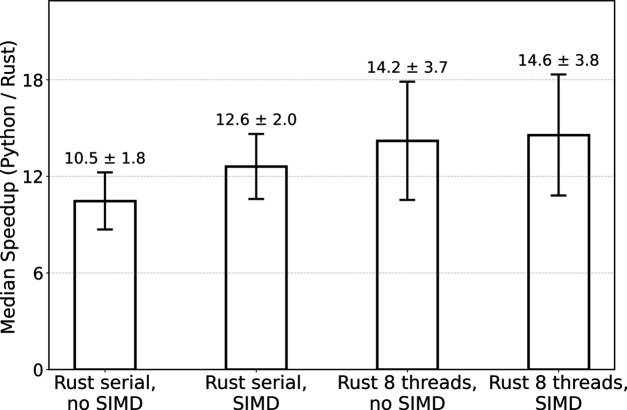
Median speedups (bars) with ± MADs (lines) for 33142 crystal structures from the MC3D, comparing different execution configurations of the Rust-accelerated XRD calculator against the original *pymatgen* implementation.

To evaluate the additional contribution of SIMD vectorization and multi-threaded parallelization, the MC3D dataset was benchmarked across four execution configurations: serial without SIMD, serial with SIMD, eight-thread parallelization without SIMD and eight-thread parallelization with SIMD (Fig. 4 ▸). The MC3D dataset consists predominantly of small structures (having between 1 and 80 atoms), for which thread initialization and SIMD setup represent a non-negligible fraction of the total calculation time, limiting the potential gain of these additional optimizations. As described above, serial execution with SIMD achieved a median speedup of 12.6× (MAD 2.0×). Compared with 10.5× (MAD 1.8×) for serial execution without SIMD (corresponding primarily to the language transition from Python to Rust), this result demonstrates about a 19% additional speedup from SIMD vectorization alone. The eight-thread parallelization without SIMD yielded a speedup of 14.2× (MAD 3.7×). Combining eight-thread parallelization with SIMD provided the highest median speedup of 14.6× (MAD 3.8×), with the SIMD effect providing only a marginal ∼3% improvement. The relatively small improvement from parallel­ization can be attributed to the absence of larger structures from the MC3D (none exceeding 80 atoms), which constrains the effectiveness of reflection-level parallelism. This trend contrasts with the COD benchmark discussed in the following section, where the presence of substantially larger structures leads to a much stronger impact of parallelization. For structures with few reflections, parallelization and SIMD overhead can partially offset computational gains, whereas for structures with large numbers of reflections both threading and SIMD provide substantially greater acceleration.

### 4.2. Performance benchmarking on the COD

**Figure 5:**
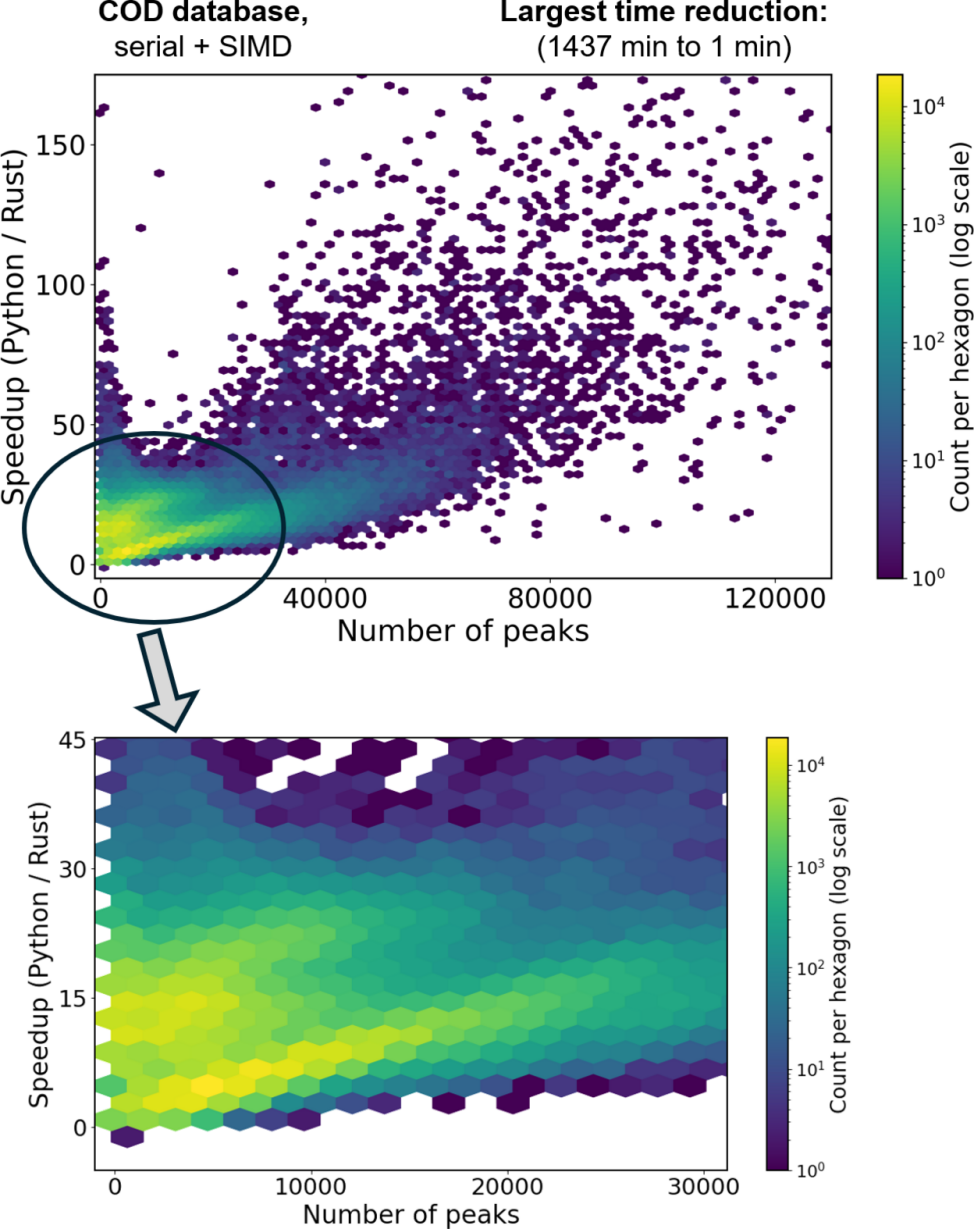
Performance comparison between the Rust-accelerated XRD calculator with serial execution and SIMD vectorization and the original *pymatgen* implementation. Benchmarks were performed on 515181 crystal structures from the COD, with powder XRD patterns computed in the 2–60° 2θ range using Mo radiation. The median speedup is 10.7× (MAD 4.2×).

For the COD dataset, benchmark performance is visualized with the *x* axis limited to approximately 130000 reflections and the *y* axis to a 160× speedup, although a small number of structures exceeded these thresholds (Fig. 5 ▸). For 2671 structures (0.5% of structures), performance was slightly worse than or comparable to the original *pymatgen* implementation. Similarly to the MC3D case, this occurred for structures with a small number of reflections, where the average computation time increased from 0.2 s with *pymatgen* to 0.3 s with the Rust implementation. For the serial Rust implementation with SIMD, the median speedup was 10.7× (MAD 4.2×), with a maximum observed reduction in runtime from 1437 to 1 min (structure ID 7201223).

**Figure 6:**
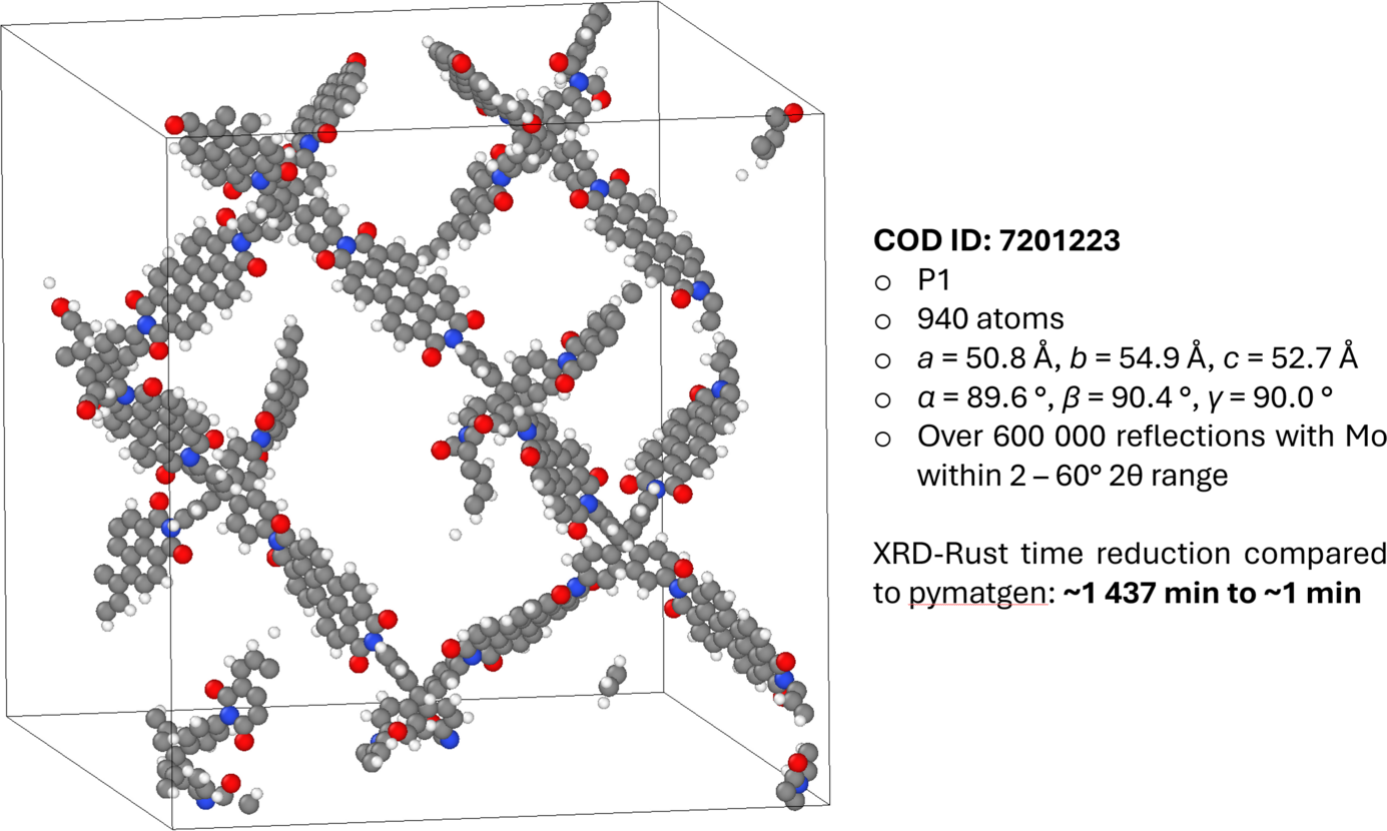
The structure from the COD (ID 7201223) having the largest time reduction (1437 to 1 min) for a powder diffraction pattern calculated by *XRD-Rust* compared with the original *pymatgen* implementation.

The maximum time reduction case, structure ID 7201223, corresponds to a large low-symmetry porous organic crystal with lattice parameters of the order of 50 Å, containing 940 atoms and yielding over 600000 reflections in the 2–60° 2θ range (Fig. 6 ▸; Trewin & Cooper, 2009 ▸). The large unit cell results in a very dense reciprocal lattice, and the consequently large number of reflections combined with the atom count leads to a very high number of individual structure-factor evaluations. In *pymatgen*, these are executed within nested Python for loops where interpreter overhead dominates the total runtime at this scale. *XRD-Rust* largely avoids this overhead through compiled machine code, while SIMD vectorization and multi-threaded execution provide additional acceleration for structures of this kind. To verify that this result was not an outlier caused by transient benchmark effects, the calculation was repeated independently and yielded a comparable speedup.

**Figure 7:**
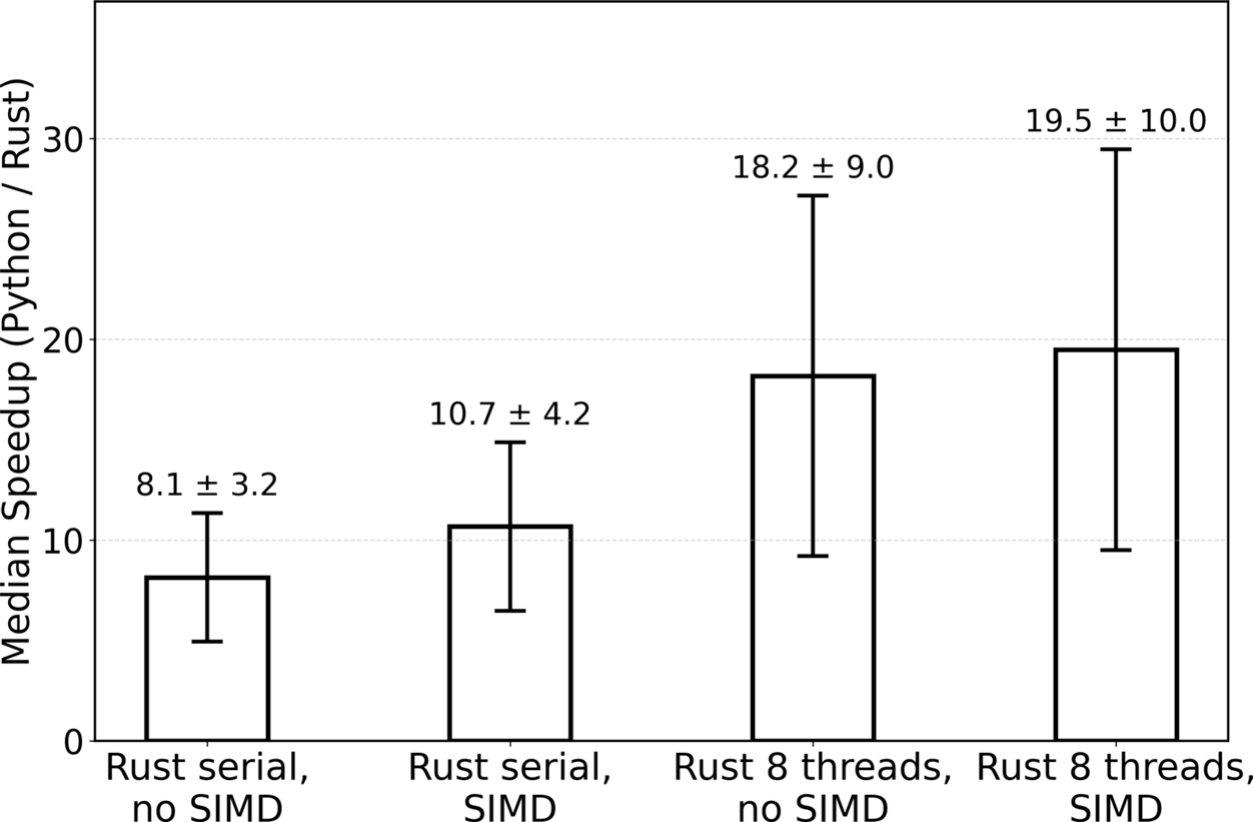
Median speedups (bars) with ±MADs (lines) for 515181 crystal structures from the COD, comparing different execution configurations of the Rust-accelerated XRD calculator against the original *pymatgen* implementation.

To evaluate the additional contribution of SIMD vectorization and multi-threaded parallelization, the COD dataset was further benchmarked across the same four execution configurations as described for the MC3D (Fig. 7 ▸). Serial execution without SIMD worsened to 8.1× (MAD 3.2×), while eight-thread parallelization without SIMD yielded 18.2× (MAD 9.0×). Combining eight-thread parallelization with SIMD provided the highest median speedup of 19.5× (MAD 10.0×). SIMD vectorization contributes an additional performance gain of approximately 32% in serial execution, whereas under eight-thread parallelization its benefit is smaller, around 7%.

**Figure 8:**
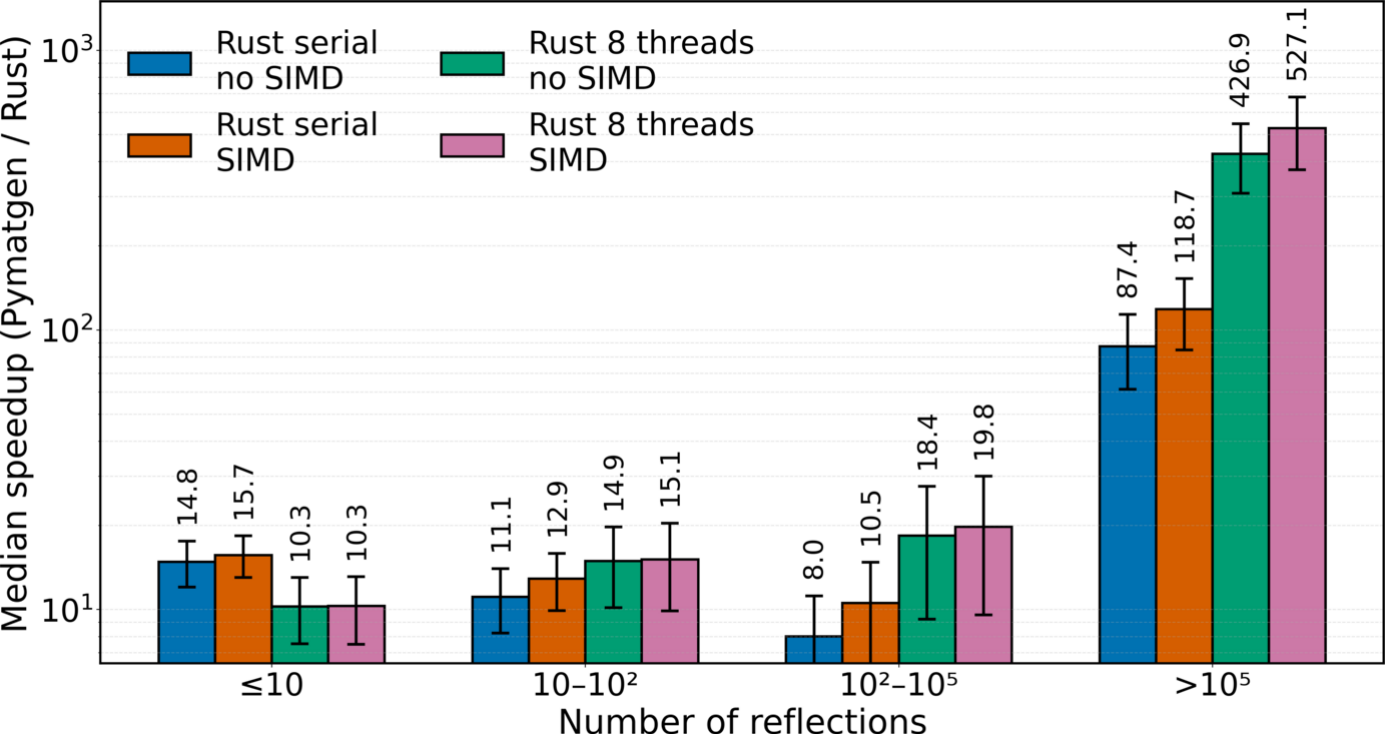
Median speedups (bars) with ±MADs (lines) for *XRD-Rust* relative to the original *pymatgen* implementation, grouped by number of reflections, illustrating the increasing effect of thread parallelization for structures with larger numbers of reflections.

To highlight the pronounced effect of thread parallelization for structures with large numbers of reflections, the speedups were grouped into four bins based on the number of reflections (Fig. 8 ▸): ≤10 reflections, 10–100 reflections, 100–100000 reflections and >100000 reflections. For the category with the fewest reflections, parallelization yielded, as expected, lower speedups (median 10.3× with SIMD) than serial execution (15.7×), as the overhead associated with parallel execution outweighs its benefit. For structures with 10–100 reflections, parallelization provided a modest improvement, increasing the median speedup from 12.9× for serial execution to 15.1×. In the third bin (100–100000 reflections), the serial median decreased slightly to 10.5×, whereas parallel execution further increased the median speedup to 19.8×. For structures with more than 100000 reflections, the effect became substantial, with median speedups increasing from 118.7× for serial execution to 527.1× with parallelization. The SIMD improvement is most pronounced for the last bin with parallel­ization, providing further speedups of about 36%.

Overall, these benchmarks demonstrate that *XRD-Rust* substantially reduces the computational cost of powder XRD simulations compared with the original *pymatgen* implementation. The performance gains arising from thread parallel­ization depend on the number of reflections: for structures with few reflections, parallelization provides only modest improvement and may be partially offset by threading overhead, whereas for structures with large numbers of reflections it yields the largest speedups. SIMD vectorization provides an additional performance benefit across configurations, ranging from a few percent to a few tens of percent. Together, these improvements enable more practical high-throughput powder XRD dataset generation for ML workflows, and they improve responsiveness in interactive applications such as *XRDlicious*. Importantly, the Python interface and notation remain closely aligned with the original *pymatgen* implementation, allowing accelerated calculations to be integrated into existing workflows with minimal code modification.

## 5. Future directions

While the present work focuses on performance optimization at the implementation level, several directions for further improvement can be considered. From an algorithmic per­spective, future work may explore more efficient approaches to structure-factor evaluation, including methods based on fast Fourier transforms (FFTs) or improved reciprocal-space partitioning strategies. From a computational perspective, additional acceleration could be achieved by extending the implementation to GPU architectures, enabling improved performance for larger systems and datasets.

## 6. Conclusions

In this work, we have presented *XRD-Rust*, a Rust-accelerated implementation of the *pymatgen* powder XRD calculator that substantially improves the computational performance of powder X-ray diffraction simulations while maintaining compatibility with existing *pymatgen*-based Python workflows. By reimplementing the computationally intensive parts of the calculation in Rust, with optional SIMD vectorization and multi-threaded execution via the *Rayon* library, significant speedups were achieved across large crystallographic datasets while preserving numerical fidelity with the original implementation.

Benchmarks on the MC3D dataset showed that the performance gain from transitioning the computational core from Python to Rust alone yielded a median speedup of 10.5× (MAD 1.8×), increasing to 12.6× (MAD 2.0×) with SIMD vectorization. Combining eight-thread parallelization with SIMD provided the highest median speedup of 14.6× (MAD 3.8×), with a maximum reduction in computation time from 40.5 to 0.9 s. For the COD dataset, serial execution without SIMD yielded a median speedup of 8.1× (MAD 3.2×), increasing to 10.7× (MAD 4.2×) with SIMD vectorization, while eight-thread parallelization achieved 18.2× (MAD 9.0×) without SIMD and 19.5× (MAD 10.0×) with SIMD. A maximum runtime reduction from 1437 to 1 min was observed for a large low-symmetry porous crystal containing more than 600000 reflections. The impact of parallelization became particularly pronounced for structures with very large numbers of reflections, where median speedups for structures with more than 100000 reflections increased from 118.7× in serial execution to 527.1× with eight-thread parallelization (both with SIMD).

These results demonstrate that *XRD-Rust* substantially reduces the computational cost of powder XRD simulations compared with the original *pymatgen* implementation, enabling more practical high-throughput dataset generation for ML-driven materials science, while also improving responsiveness in interactive applications such as *XRDlicious* and keeping the original *pymatgen* notation.

## 7. Code availability

The source code and its documentation are publicly available on GitHub at https://github.com/bracerino/xrd-rust and can be installed from the Python Package Index (PyPI) (https://pypi.org/project/xrd-rust) using pip install xrd-rust. The package is distributed for Linux, macOS and Windows platforms. The *XRD-Rust* package has been employed into an interactive online application (https://xrdlicious.com) to speed up calculations of powder XRD patterns.
